# The impact of generational change and retirement on psychiatry to 2025

**DOI:** 10.1186/1472-6963-7-141

**Published:** 2007-09-04

**Authors:** Susan L Fletcher, Deborah J Schofield

**Affiliations:** 1 Northern Rivers University Department of Rural Health, University of Sydney, PO Box 3074, Lismore, NSW 2480, Australia

## Abstract

**Background:**

Australia is currently experiencing widespread shortages of psychiatrists. The changing nature of the workforce and increasing demand mean that these shortages are unlikely to ease. This study aims to identify demographic change and retirement patterns of the Australian psychiatry workforce from 1995 to 2003, and the implications of those changes for future workforce planning.

**Methods:**

Data from the Australian Institute of Health and Welfare (AIHW) Medical Labour Force Survey from 1995 to 2003 is used to examine ageing of the psychiatry workforce and attrition of psychiatrists aged 50 years and over. Future attrition from the workforce is projected to 2025.

**Results:**

Sixty two percent of psychiatrists practicing in the year 2000 are predicted to have retired by 2025. Most psychiatrists continue to work until late in life, with only 18 per cent retiring before age 65. The psychiatry workforce aged significantly between 1995 and 2003 (p < 0.001), with men older than women in both years. A reduction in hours worked by psychiatrists reflects both the increasing proportion of females and the older members of the profession reducing their hours in preparation for retirement.

**Conclusion:**

The impact of ageing of the workforce may be more immediate for psychiatry than for some other health professions. With the growing proportion of females and their typically lower workforce participation, more than one younger psychiatrist will be required to replace each of the mostly male retirees.

## Background

Psychiatrists are in short supply across Australia, with the situation particularly critical in rural and remote areas [1-3] and in some subspecialties, including child and adolescent and old age psychiatry [1,2]. While psychiatrist numbers have risen in recent years [1,2,4-6], the changing nature of the workforce means that existing shortages are unlikely to be overcome by increased numbers alone.

The Australian psychiatric workforce is ageing [1,2,7], with 40 percent of practitioners aged over 55 in 2003 compared to 33 percent in 1999 [4]. Of particular concern is the large baby boomer cohort, which currently makes up around 57 per cent of the Australian health workforce [5]. With the oldest of the boomers now in their sixties, the impending retirement of this group is likely to place additional pressure on an already strained profession. Psychiatrists have previously been found to reduce their working hours as they head towards retirement [4,6]. Thus the effect of baby boomer ageing on workforce capacity is likely to be substantial even before they leave the workforce.

Although psychiatry has one of the highest female participation rates of the medical specialties [7], it has historically been a male dominated profession [6,9,10]. While that remains the case, women have been joining the specialty in ever increasing numbers [1,2,8]. In 2005, women made up around one third of psychiatrists in Australia and half of psychiatry trainees [1], with a higher proportion of women in the younger age groups than in the workforce as a whole [1,2,9]. As female psychiatrists tend to work fewer hours than their male counterparts [1,12,13], this younger female workforce may be limited in its capacity to replace the mostly male older cohorts.

In terms of the demographics and growth of the workforce, the patterns in Australian psychiatry are similar to those observed in other countries. The United States and Canada have both seen a rise in the number of psychiatrists [14-16] and proportion of women [12,16-18] in recent times. Women in the United Kingdom are also joining the profession in greater numbers [10]. The U.S. workforce is ageing [12,14,15], and as in Australia, older psychiatrists are reducing their working hours [11]. The trend for physician shortages to be more severe outside urban areas also exists in Canada [12], New Zealand [13] and South Africa [14].

With the changing demographics of the psychiatry workforce, existing shortages are unlikely to be overcome in the near future. Despite predictions of shortage as a result of retirement of the older cohorts, little information about the actual retirement rate of health professionals exists according to a recent World Health Organisation report, and the methods of analysis used tend to assume retirement at an arbitrary age [15]. This study examined trends in the work practices, ageing, and retirement patterns of psychiatrists. It overcomes some of the limitations of previous studies by estimating the wide variation in the age of retirement and numbers of retirees at different ages.

## Methods

The methods used in this study are similar to those described by Schofield and Beard [16]. Previously unpublished data on the demographic characteristics (age, sex and hours worked) for psychiatrists in five year age bands were obtained from the Australian Institute of Health and Welfare (AIHW) Medical Labour Force Survey for the years 1995 to 2003. Survey responses are weighted to psychiatrist registration numbers, and the data is therefore an estimate of total psychiatrist numbers in each survey year.

As individuals cannot be followed from one survey to the next, age groups were followed as cohorts. Boundaries were applied which aligned with the five year age groups of the data. The cohorts for the current study were defined as follows: Pre-depression (born before 1929), War and Depression (born 1929–1945), Older Baby Boomers (born 1946–1956), Younger Baby Boomers (born 1957–1964), Generation X (born 1965–1975) and Generation Y (born after 1975). The data within these generational cohorts was used to examine demographic trends in the psychiatric workforce.

Workforce attrition was calculated as psychiatrists ceased working because of factors such as retirement, ill health, or change of career. Cumulative attrition was calculated for each sex for each of the 5 year age bands over 50 and were defined as the percentage of psychiatrists in each of these age groups who had left the workforce in the two five year periods between 1995 and 2005:

CAR = 1 - N_ti_/N_t1_

Where CAR = Cumulative attrition rate,

N = number of people,

ti = year in series, and

t1 = first year in series (1995)

As data was only available for 9 years up to 2003, attrition to 2005 was calculated for each older age and sex group as CAR_95–05 _= CAR_00–03 _× 1 2/3 + CAR_95–00_.

These attrition rates were then applied to psychiatrists to project workforce attrition over the next 20 years. The assumption underpinning the model was that historical rates of retirement will be reflected in future cohorts.

Data analysis was undertaken using SAS version 9.1. All tests were conducted at a 0.01 level of significance, with χ^2 ^tests used to carry out tests of association between categorical variables.

## Results

Between 1995 and 2003 Australian psychiatrist numbers increased by approximately 27 per cent, from 2389 to 3026 (Table 1). Distribution of psychiatrists among the cohorts remained relatively stable. In both years, the baby boomer groups accounted for over half of the workforce. However in 2003 the oldest two cohorts accounted for 20 per cent of psychiatrists compared to 36 per cent in 1995, while generation X had increased from 6 per cent to 24 per cent as younger psychiatrists entered the workforce.

**Table 1:** Definition and size of cohorts

		Number of psychiatrists (% of total workforce)	
Cohort	Birth years	1995	2003

Pre-Depression	Before 1929	244 (8%)	83 (3%)
War and Depression	1929–1945	661 (28%)	513 (17%)
Older Baby Boomers	1946–1956	708 (30%)	738 (24%)
Younger Baby Boomers	1957–1964	630 (26%)	864 (29%)
Generation X	1965–1975	143 (6%)	720 (24%)
Generation Y	After 1975	0	108 (4%)
Total workforce		2389	3026

Generation Y, the oldest aged 28 in 2003, had not completed its entry into the workforce in 2003. Similarly, not all of Generation X (the youngest aged <25 in 1995) had entered the workforce in 1995.

**Figure 1:**
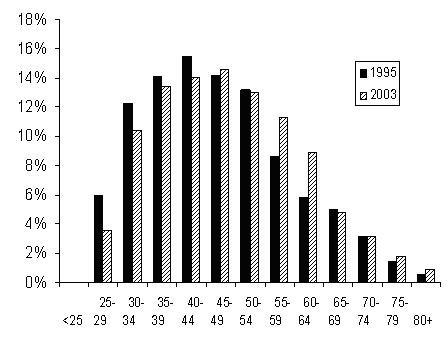
Age distribution of psychiatrists, 1995 and 2003.

The psychiatry workforce has aged significantly (p < 0.01), as shown in Figure 1. In 1995, 38 per cent of psychiatrists were aged 50 years or more. That figure rose to 44 per cent in 2003.

While both sexes showed this pattern of ageing, male psychiatrists were older than their female counterparts in both the first and last survey years (p < 0.01), and in 2003 made up 74 per cent of psychiatrists aged 50 years or more. Forty three per cent of men were aged over 50 in 1995 and 50 per cent in 2003, compared to 23 and 33 per cent of women respectively.

### Proportion of females in the workforce

**Figure 2:**
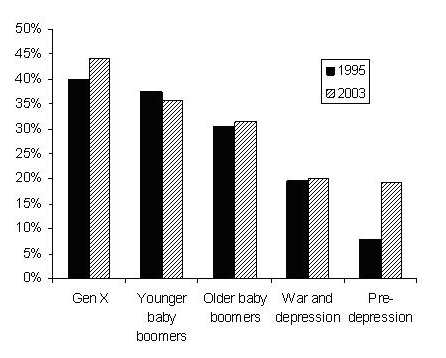
Proportion of females in psychiatry, 1995 and 2003.

While female psychiatrists continue to be outnumbered by males, the gender divide is slowly closing. In 2003, 35 per cent of the workforce was female compared to 28 per cent in 1995. Women have greater representation in the younger age groups, accounting for 44 per cent of generation X psychiatrists, compared to one fifth of those in the two older cohorts (Figure 2).

The feminisation of psychiatry looks set to continue. While generation Y had not completed its entry into the workforce in 2003, 70 per cent of the small number who were working in that year (108) were female.

### Workforce attrition

Attrition from 1995 to 2005 was calculated for all psychiatrists aged over 50. Table 2 shows that of psychiatrists aged 55–59 in 1995, 46 per cent had left the workforce by age 65–69. For psychiatrists aged 70–74 in 1995, 37 per cent retired within 5 years and 80 per cent by the time that group was aged over 80. Women tended to retire in larger proportions in the older age groups but the opposite was true for the younger psychiatrists in this analysis. However, there were relatively small numbers of older female psychiatrists (eg 19 aged 65–69 in 1995) and results should interpreted with this in mind.

**Table 2:** Cumulative attrition rates of older psychiatrists, 1995 to 2005

Year	50–54	55–59	60–64	65–69	70–74
Male psychiatrists					
1995 numbers	243	169	119	102	75
2000	11%	0%	19%	40%	54%
2005	18%	46%	35%	61%	91%
Female psychiatrists					
1995 numbers	72	38	20	19	0
2000	0%	0%	25%	53%	*
2005	18%	45%	58%	79%	*
All psychiatrists					
1995 numbers	315	207	139	121	75
2000	8%	0%	21%	41%	37%
2005	18%	46%	38%	63%	80%

Age groups can be followed down the columns of the table. For example, 8% of the 315 psychiatrists aged 50–54 years in 1995 had left the workforce by the year 2000, when they were aged 55–60.

Source: AIHW Medical Labour Force Survey, 1995 to 2003. Calculation of cumulative attrition rates for each cohort: CAR = 1 - Nti/Nt1 where CAR = Cumulative attrition rate, N = number of people, ti = year in series and t1 = first data year in series (1995). Calculation of attrition from 2000 to 2005 = CAR_2000–2003 _* 1 2/3.

* There were no female psychiatrists aged 70–74 in 1995, thus attrition could not be calculated for this group.

**Figure 3:**
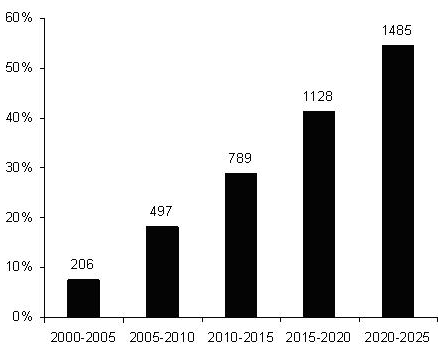
Five year cumulative attrition rates for psychiatrists projected from 2005 to 2025.

The total attrition rates (men and women combined) in table 2 were applied to younger psychiatrists to project their attrition from the workforce from 2000 to 2025 (Figure 3). Total attrition was used rather than sex specific rates as the small number of older female psychiatrists made figures unreliable for long term projections. Eighteen per cent of psychiatrists working in the year 2000 are expected to retire by 2010, with that figure growing to around one third by 2015. By 2026, 55 per cent of the 2000 workforce will no longer be practicing, if attrition rates remain steady at 1995–2005 levels.

### Hours worked

**Figure 4:**
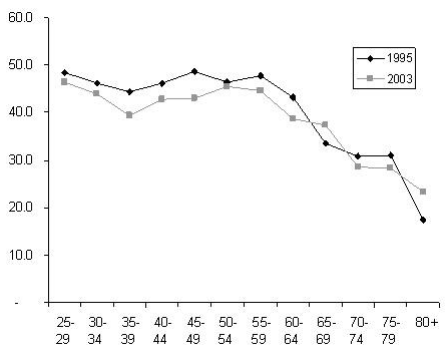
Average weekly hours worked by psychiatrists in 1995 and 2003, by age.

There is a shift towards psychiatrists working fewer hours, with average weekly hours dropping from 45.0 in 1995 to 41.8 in 2003. This shift is partly due to the growing proportion of psychiatrists in the older age groups, as the older members of the profession tend to work fewer hours than their younger counterparts (Figure 4).

However, younger psychiatrists in generations X and Y are also working fewer hours than their predecessors did at the same age. In fact, in 2003, generation X were on average working fewer hours than either of the baby boomer cohorts who are moving towards retirement.

**Figure 5:**
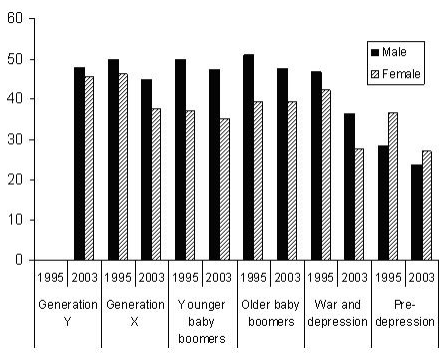
Average weekly hours worked by male and female psychiatrists in 1995 and 2003.

The trend towards shorter working weeks is also likely to reflect the growing number of women in psychiatry. Women have traditionally worked fewer hours than men, and in 2003 worked an average of 37 hours per week compared to 44 for men. This gender difference has remained relatively stable since 1995, when women worked 39 hours and men worked 47. Figure 5 shows the hours worked by men and women in each cohort in 1995 and 2003. Differences between the two sexes are particularly evident among the younger baby boomers, who were aged 30–39 in 1995 and 40–49 in 2003. However, the most striking finding is that both women and men in most cohorts have lower working hours in 2003 than in 1995. The longer hours worked by women than men in the pre-depression cohort is likely to reflect small sample size and should be interpreted with caution.

## Discussion

Existing workforce shortages in psychiatry are likely to be exacerbated as the older members of the profession retire. Ageing of the workforce may have a greater and more immediate impact on psychiatry than some other health care sectors. Around three quarters of psychiatrists are aged over 40, compared to 65 per cent of all medical specialists and 58 per cent of general practitioners [16]. Future workforce capacity may be further limited by the trend towards shorter working weeks and the growing proportion of female psychiatrists.

While psychiatrists have previously been predicted to retire at age 70 [2], our data shows that the majority (62 per cent) remain in the workforce beyond that point. Psychiatrists retire later than the Australian population in general (where the average age of retirement for men is 58 years, for persons aged 45 or older) [17], and many do not expect to retire at all [6]. As clear retirement guidelines in psychiatry do not exist, beyond the need to maintain competence [6], psychiatrists may choose to keep working well into old age. Fears about losing their professional role, letting patients down, and denial of waning competence [6] may also lead psychiatrists to remain in the workforce longer than the average Australian. An additional barrier to retirement for psychiatrists in private practice can be the need to find a replacement to take over their practice [6].

In addition the relatively late retirement of psychiatrists may be financially driven. Most older psychiatrists are male, have higher than average earnings [5], and are more likely to work in private practice than their younger counterparts [7]. Without compulsory superannuation and active financial planning, an awareness of the need for retirement savings may not be acquired until later in life. After the realisation that they face a significant fall in income after retirement, many psychiatrists may work for longer than they had planned in an effort to add to their savings. The trend towards earlier retirement of psychiatrists in the public sector [6] may be partly explained by their compulsory superannuation contributions.

The effect of recent changes to Australian superannuation laws on the retirement of psychiatrists is yet to be seen. The removal of a 15 per cent tax on retirement from a super scheme after the age of 60 years [18] may encourage some of the 8 per cent of psychiatrists who have in the past retired before 60 to remain in the workforce. However, it could also allow some of the 92 per cent who work beyond 60 years to retire early. As the benefits of superannuation are becoming more widely understood, younger psychiatrists could be expected to be more financially prepared for retirement and consequently to leave the workforce earlier than their predecessors.

The late retirement age of psychiatrists may be expected to somewhat mitigate the effect of ageing on the psychiatry labour force. However, as this and previous studies have found, psychiatrists tend to reduce their hours as they age [7,9,12]. Therefore, the 44 per cent of Australian psychiatrists currently aged 50 or more are likely to decrease their workforce participation as they head towards retirement, reducing the effective supply of the workforce even while they remain in it.

The effect on workforce capacity once these older psychiatrists do begin to retire will be substantial. Previous authors have noted the importance of increasing recruitment of medical students into psychiatry in order to sustain the specialty [11]. Twenty one per cent of the 2000 workforce is predicted to retire between 2005 and 2015. This equates to a loss of 583 psychiatrists. According to the Medical Training Review Panel (MTRP), between 2000 and 2005 an average of 83 new fellows per year entered the profession (ranging from 70 to 109) [19]. This increase on the 72 new psychiatrists reported by the Australian Medical Workforce Advisory Committee (AMWAC) to enter the workforce annually throughout the 1990s [2] may be partially due to differences in classification; AMWAC counted only Australian training program graduates while the MTRP data includes Australian graduates and new fellows who completed their training overseas. It is worth noting that since the AMWAC report in 1999, there has been no government review of the psychiatry workforce and training numbers in Australia.

At the level reported by the Medical Training Review Panel, 830 young psychiatrists could be expected to enter the workforce over the ten years from 2005. While the net gain of 247 psychiatrists may go some way towards easing the strain on the profession, current shortages may not be entirely overcome, particularly in the context of population growth, increasing demand and rising proportions of females (who work fewer hours) in the younger age groups.

The gap left by older male retirees may not be completely filled by young females, whose lifetime participation in the workforce is generally lower than that of males. Women are more likely to work part time and retire earlier than males [9,20,21]. They are also more likely to take time out of the workforce to have and raise children. Factoring in these gender differences will be crucial to future workforce planning [10].

It is important to note that the projections calculated in this study assume retirement rates will remain steady in the future. With the rising proportion of women and their typically earlier retirement, the 55 per cent of the workforce predicted to retire by 2025 may in fact be a conservative estimate.

While the ability of the psychiatry workforce to deliver services may decline in coming years, the demand for those services is likely to rise[21]. Current estimates suggest that around one fifth of the Australian population will experience mental illness [2,22] and as the population grows, so will the absolute numbers of Australians suffering such problems. Existing shortages in old age psychiatry [1,2] are likely to worsen with the ageing of the population. The stigma surrounding mental illness is slowly decreasing thanks in part to its rising profile in the popular media, including recent advertising campaigns raising awareness for illnesses such as depression and schizophrenia. As mental health becomes less of a taboo subject, more people may seek treatment and the strain on psychiatrists will continue.

While increasing student numbers is vital for the long term sustainability of the profession, the RANZCP's training program takes most students more than six years to complete [2] and thus the effect of additional trainees would not be seen in the workforce for the best part of a decade. Other options for increasing workforce capacity and providing more effective mental health services must therefore be explored. One possibility is to recruit more overseas trained psychiatrists. At present, about 7 such specialists enter the permanent workforce each year [1]. A global shortage of psychiatrists, however, means that this is unlikely to be an effective long term solution [2,15].

Another option is for psychiatrists to delegate some of their duties where best practise treatment would not be compromised. Again, this cannot be the sole solution to the current situation as Australia is also experiencing a shortage of other mental health workers, particularly mental health nurses [1]. General practitioners are the first port of call for the majority of individuals who seek help for mental health issues [22,23], with psychological problems accounting for around 8 percent of all problems managed by GPs [24]. Past difficulties in providing quality mental health care in the general practice setting are being overcome by initiatives such as Better Outcomes in Mental Health Care which provides GPs with education and training to increase mental health skills, special Medicare items for longer consultations, and access to allied health and psychiatrist support [21,22].

Recent changes to Medicare, Australia's universal health insurance program, mean that some patients referred to a psychologist are now eligible to receive some reimbursement. The full impact of these changes is yet to be seen but they may lead GPs to refer more to psychologists, thus reducing demand for psychiatrists and easing the pressure on the increasingly female and part time psychiatry workforce. However, psychologists may still not be an option for many Australians, as the differences in Medicare rebates between the two professions are substantial. An initial consultation with a psychiatrist currently attracts a $340 subsidy, while 50 minutes with a Medicare registered psychologist will see the patient refunded $75; the relativity in the rebate does not reflect the difference in the prices faced by consumers. In addition, although psychologist services are partly covered by many private health insurance plans, those likely to be most in need of this coverage are also the people least likely to be able to afford private health insurance.

Although psychologists in some states of the U.S. have been granted prescription privileges [25], at this stage such legislation has not been enacted in Australia and thus psychiatrists remain unique in their ability to provide both pharmaco- and psychotherapy. It may be, therefore, that psychologists and GPs can ease the burden on psychiatrists by treating affective, sleep, and other high prevalence but relatively low severity disorders, allowing psychiatrists to focus on schizophrenia, personality disorders, and others which require more comprehensive treatment.

## Conclusion

In summary, psychiatry in Australia is currently experiencing serious labour force shortages, which are likely to worsen as almost two thirds of psychiatrists are predicted to retire by 2025. Increasing demand and the changing nature of the workforce will further limit the ability of psychiatrists to provide sufficient services. To overcome the problem, a number of solutions must be investigated including substitution of other health care providers, recruitment of overseas trained psychiatrists and most importantly for the long term, increasing student numbers. Any of these alone is unlikely to improve the capacity of psychiatrists in Australia to meet demand.

## Competing interests

The author(s) declare that they have no competing interests.

## Authors' contributions

SF drafted and edited the manuscript and analysed the data. DS conceived of the study and edited the manuscript. Both authors read and approved the final manuscript.

## Pre-publication history

The pre-publication history for this paper can be accessed here:


